# Comparative Analysis between Conventional Acrylic, CAD/CAM Milled, and 3D CAD/CAM Printed Occlusal Splints

**DOI:** 10.3390/ma16186269

**Published:** 2023-09-19

**Authors:** Cristian Abad-Coronel, Carolina Ruano Espinosa, Sofía Ordóñez Palacios, César A. Paltán, Jorge I. Fajardo

**Affiliations:** 1 CAD/CAM Materials and Digital Dentistry Research Group, Faculty of Dentistry, Universidad de Cuenca, Cuenca 010204, Ecuador; 2 Faculty of Dentistry, Universidad de Cuenca, Cuenca 010204, Ecuador; carolinaa.ruano@ucuenca.edu.ec (C.R.E.); grace.ordonezp@ucuenca.edu.ec (S.O.P.); 3 New Materials and Transformation Processes Research Group GiMaT, Universidad Politécnica Salesiana, Cuenca 010105, Ecuador; cpaltan@ups.edu.ec (C.A.P.); jfajardo@ups.edu.ec (J.I.F.)

**Keywords:** occlusal splint, digital dentistry, CAD/CAM materials, fracture resistance, 3D printing

## Abstract

The development of digital technologies has allowed for the fabrication of new materials; however, it makes it difficult to choose the best methods to obtain occlusal splints with optimal properties, so it is essential to evaluate the effectiveness of these materials. The aim of the study is to compare the fracture resistance of occlusal splints made of different materials after thermo-mechanical aging. Methods: A total of 32 samples were made from 4 materials (two 3D printed polymeric materials, a PMMA disc for CAD/CAM, and a conventional heat-cured acrylic resin); subsequently, the fracture test was performed using the load compression mode applied occlusally on the splint surface. Statistical analysis: Four statistical tests were used (Shapiro-Wilk, Levene’s test, ANOVA, and Tukey’s HSD test). Results: The following study showed that there are differences in fracture strength among the four materials investigated, where the highest strength was observed in the milled splint, with a mean of 3051.2 N (newton) compared to the strength of the flexible splint with 1943.4 N, the printed splint with 1489.9 N, and the conventional acrylic splint with 1303.9 N. Conclusions: The milled splints were the most resistant to fracture. Of the printed splints, the splint made with flexural rigid resin withstood the applied forces in acceptable ranges, so its clinical indication may be viable. Although the results of this research indicated differences in the mechanical properties between the CAD/CAM and conventional fabrication methods, the selection may also be influenced by processing time and cost, since with a CAD/CAM system there is a significant reduction in the production time of the splint material.

## 1. Introduction

The occlusal splint is a device that is characterized by reducing the signs and symptoms related to temporomandibular disorders (TMD), reducing sensitivity to palpation, providing protection against tooth wear, improving jaw movements and mouth opening, and achieving neuromuscular balance by stabilizing occlusion [1]. Among its most important functions are the following: protecting associated structures and teeth from bruxism; mitigating proprioception of the periodontal ligament; decreasing cellular hypoxia; positioning the condyle within the glenoid cavity in a stable musculoskeletal position; and providing diagnostic information about clenching or grinding, such as allowing the dentist to observe while the patient wears it [2]. The splint should provide uniform, bilateral occlusal contacts of equal intensity without altering the resting position of the mandible or dental occlusion. In addition, it should be retained, adjusted, and stabilized without generating hypersensitivity in the dental structure [3]. It should be noted that the effectiveness of the occlusal splint is associated with the frequency of follow-up and the precise occlusal adjustment made by the professional in the dental practice [4]. Intraoral devices should be made of materials capable of simulating the micro-hardness and modulus of elasticity of dentin, which range from 250 to 800 MPa and 10 to 20 GPa, respectively. These values compensate for the stiffness of enamel with the ability to cushion masticatory impacts, have good wear behavior, and have an acceptable surface finish. This would prevent changes in the appliance such as discoloration, micro-porosity, early biofilm formation, and occlusal instability [5,6]. Below are the different types of splints according to the way they were made: lost-wax enameled, milled, and 3D printed.

The conventionally made splint contains clear, self-curing methyl-methacrylate (MMA powder/liquid), which is obtained by making an impression, casting in plaster models, designing, and inter-occlusal wax registration [7,8]. Among the advantages of this type of splint are its ease of fabrication and low cost compared to other treatments such as complete rehabilitation or orthodontic treatment. However, it has some disadvantages, such as polymerization shrinkage, susceptibility to fractures, dimensional instability, and the long delivery time of the laboratories [9].

The use of CAD/CAM systems (computer-aided design and manufacturing) has innovated the dental processes from conventional to digital, allowing the registration of the data obtained from the two jaws made with the intraoral scanner by means of a software program and going on to the making of splints through subtractive (milling) or additive (3D printing) methods. This type of technology has demonstrated greater benefits in terms of dimensional stability, speed, better retention, and greater reproducibility [10,11].

Splints produced by 3D printing can be made using CAM, adding material layer by layer to form a three-dimensional model with a high degree of geometric complexity. It should be noted that this type of additive manufacturing offers great design flexibility, a minimum amount of wasted material, efficiency, and fast, accurate results.

The most well-known 3D printing processes are stereolithography (SLA), material jetting (MJ), digital light processing (DLP), fused deposition modeling (FDM), and selective laser sintering (SLS) [12]. The dimensional accuracy of 3D printing is given by three types of xyz segments, of which the x-y axes are responsible for modeling the object by depositing the material, while on the z-axis the layers of the object are developed [13].

1. Stereolithography (SLA) is considered the first 3D printing technology, and it is a process that uses laser light to convert a resin from a liquid to a solid state. Each layer formation generates strong models with high detail quality, a good surface finish, and a high level of precision. SLA involves post-processing time for the removal of resin that remained uncured, implying additional cost when using large objects with an accuracy of 35–40 μm, and is recommended for the production of implant drill guides [14].
2. Digital Light Processing (DLP) uses a high-power light-emitting diode (LED) as a light source to polymerize liquid resins and contains a digital micro-reflector (mirror arrangement). Each mirror represents a pixel, and the emitted light is refracted by this micro-reflector. Detailed processing has a faster production time than stereolithography because the entire layer can be built with a single irradiation and each layer is built independently according to its shape, so small objects with high-quality details can be obtained, as well as smooth and polished surfaces with an accuracy of 50–55 μm. DLP is used for the production of surgical guides, aligners, bridges, and splints [15,16].
3. Material Jetting (MJ) consists of depositing the acrylic material in the form of droplets that polymerize through ultraviolet radiation until a three-dimensional model is built layer by layer. MJ allows the combination of several materials so that they can form objects with different properties. This type of technology allows for a good surface finish and high resolution, but it also has a long printing time, very thin layer thicknesses, and reduced mechanical properties, which lead to an approximate precision of 25 to 30 μm, making it suitable for anatomical models [16,17].
4. Fused Deposition Modeling (FDM) is a fast and low-cost printing method in which the nozzle heats and melts the thermoplastic material that is in a filamentous state. The material is extruded and solidified by accumulating layers to manufacture the model with an accuracy of 30–40 µm. This technique can generate rough surfaces and is therefore only suitable for study models or aligners due to its low precision [16].
5. Selective Laser Sintering (SLS): in this type of technology, particles of resin powder are bonded together to create a solid model through a CO_2_ laser beam with a repetitive process, creating one layer at a time until the process is completed with an accuracy of 45–50 μm. It is used for the fabrication of metal crowns, bridges, partial dentures, and orthodontic appliances [13,16].

**Materials for 3D dental printing:**

The most suitable materials for 3D printing are thermoplastic polymers that are made of filaments, which are heated and conform to specific structures. The aforementioned material has several advantages based on having a better manufacturing resolution that allows for good mechanical resistance, smooth surfaces, and good chemical bonds, which leads to better biocompatibility between the material and the patient [18]. 

According to the SprintRay, Los Angeles, CA, USA manufacturer’s specifications, the resins for occlusal splints are:

- **Resin Splint**

Flexural strength: ≥105 MPa
Elastic module: ≥2452 MPa
Printing time: 35 min
Suggested layer thickness: 50–100 μm

- **Resin Night-Guard Flex**

Flexural strength: ≥118 MPa
Elastic module: ≥2452 MPa
Printing time: 44 min
Suggested layer thickness: 100–150 μm

Subtractive fabrication of splints is a method of cutting material from a prefabricated disc or block whose milling depends on the number of axes of the machine, which can be three, four, or five axes.

**3-axis device**

The three-axis device has movements in three spatial directions, which are X, Y, and Z. During the milling processing in the dental area, the 3-axis devices can rotate the component by 180°. The advantages of using this machine are a short milling time and simplified control. In addition, they are usually less expensive than 4- and 5-axis machines. They are recommended for the fabrication of veneers, posterior indirect restorations, fixed dental prostheses, and crowns [19].

**4-axis device**

In a 4-axis machine, in addition to the X, Y, and Z planes, the material is supported by a tension bridge on which it can rotate infinitely. As a result, it is possible to adjust the construction bridge on which the milling spindle is supported with the same block, achieving a vertical displacement and saving material and processing time. This type of device is used for indirect posterior restorations [19].

**5-axis device**

In the 5-axis machine, as well as the three spatial axes X, Y, and Z and the rotation of the tension bridge (A), it is possible for the machining spindle to also rotate and generate another axis of rotation (B). This makes it possible to machine complex geometries with subsections, such as fixed bridge frameworks with several pontics, abutments, therapeutic splints, complete dentures, or implant-supported bridges [19].

**Material for milling dental splints:**

The material commonly used for the fabrication of milled splints is PMMA. PMMA CAD/CAM discs, in their production method, use high polymerization pressure and temperature values, developing long polymer chains with reduced intermolecular distances, allowing the material to be less porous and with less residual monomer. Therefore, it is ready to go through a bright polishing process after milling without the need for further post-production [20]. 

The PMMA-milled splint has advantages such as greater patient comfort due to its slim design with thicknesses down to 0.3 mm and good optical properties due to good color stability [20].

According to the manufacturer’s specifications, the properties of the PMMA disc (ProArt CAD Disc) are:

- Flexural strength ≥ 100 MPa.
- Modulus of elasticity ≥ 2800 MPa.
- Hardness ≥ 140 MPa.
- Water absorption ≤ 40 μg/mm^3^.
- Solubility ≤ 7.5 μg/mm^3^.

Following the background described above, the purpose of this study was to evaluate the microstructural characteristics of occlusal splints using digital CAD/CAM and conventional workflows and compare labor time and cost. The null hypothesis of this study was that there would be no difference between the fracture strengths of different materials for the fabrication of occlusal splints.

However, studies obtained on the comparison of the resistance to fracture between different materials for the fabrication of occlusal splints using subtractive and additive techniques are very limited. That is why this study aimed to compare the resistance of fracture materials for the fabrication of occlusal splints.

## 2. Materials and Methods

### 2.1. Sample Fabrication

**Figure 1:**
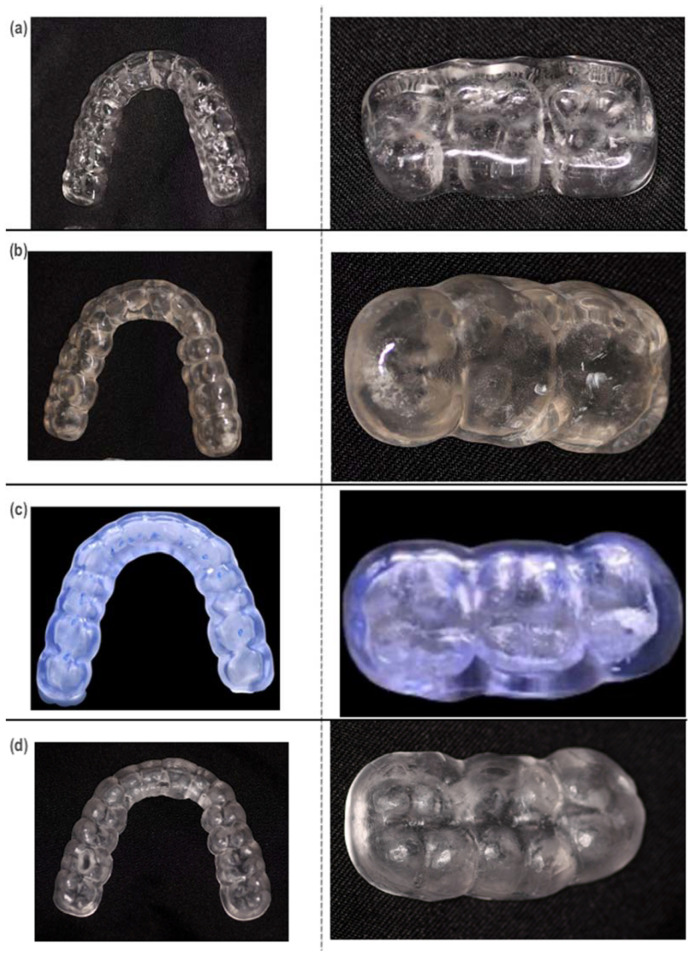
(**a**) Acrylic splint sample. (**b**) 3Dprinted splint sample (resin splint). (**c**) 3D-printed splint sample (resin flex). (**d**) Milled splint sample.

**Figure 2:**
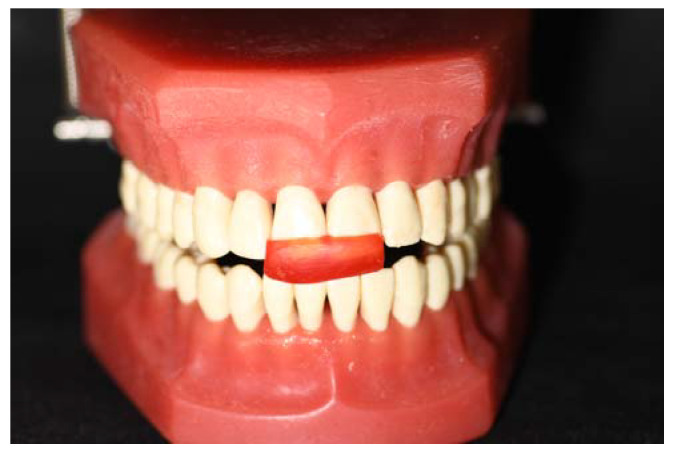
Jig and verification of the occlusal space to standardize the splint thickness.

A total of thirty-two specimens were fabricated on a one-splint model of a three-piece upper (second premolar, first molar, and second molar) (Figure 1). The specimens were divided into four groups (n = 8): (1) acrylic splint (VERACRIL^®^, OPTI-CRYL^®^ HEAT-CURING ACRYLIC, Antoquia, Colombia); (2) printed splint (a resin splint from Sprintray, Los Angeles, CA, USA); (3) flex printed splint (a resin nightguard flex from Sprintray, Los Angeles, CA, USA); and (4) milled splint (ProArt CAD Splint from Ivoclar, Schaan, Liechtenstein). The upper and lower arches of a dental model were scanned using the scanner (Primescan 2.0, Dentsply-Sirona, Charlotte, NC, USA); a digital impression of the typodont was obtained and subsequently digitized in a design software (InLAB 22.2, Dentsply-Sirona, New York, NY, USA); and the splints were standardized in a three-piece design that simulates the intraoral design conditions, establishing thicknesses of 2 mm (Figure 2) both in the occlusal and in the supporting walls, and transferred to a CAM software (InLab CAM, 22.2, Dentsply-Sirona, New York, NY, USA) corresponding to a 5-axis laboratory milling machine (MCX5, Dentsply-Sirona, Charlotte, NC, USA) (n = 8). The same design was transferred to the CAM software of the 3D printer (SprintRay Pro-95, Los Angeles, CA, USA), establishing 100 µm as the suggested thickness according to the manufacturer, and the samples were printed (n = 16), which went through a post-production process using an automated multi-stage wash system (Pro Wash/Dry, Sprintray, Los Angeles, CA, USA) and an automated light curing system (ProCure 2, Sprintray, Los Angeles, CA, USA).

Additionally, conventional splints were made as a control group (n = 8) in the research to determine if they are efficient and accurate, as well as the digital workflow, so the impression is taken in the traditional way: assembly in the articulator, manufacture of the jig, placement of acetates and waxing of the plate, the process of moulding to obtain the splint, and subsequent post-production. For postproduction, all samples were polished with grain discs with sizes in decreasing order: coarse (95 µm), medium (50 µm), and fine (5 µm) with continuous wetting for 1 min and finished with the use of cotton hair wheels with pulverized pumice and polishing paste.

Once finished, all samples were subjected to a computerized thermocycling process in a unit for the effect (ThermocyclerTM, SD Mechatronik, Feldkirchen-Westerham, Germany) at 5000 cycles with extreme temperatures of 5 °C and 55 °C in distilled water (residence time: 25 s, pause time: 10 s).

### 2.2. Fracture Resistance Test

For this purpose, one model of a three-piece upper metal die (second premolar, first molar, and second molar) was fabricated from a beryllium-free nickel-chromium alloy (Wirona, Bego Bremer Goldschlägerei, Bermen, Germany), and a universal testing machine (Shimadzu AGS-X series Universal Testing Machine; Tokyo, Japan) was used for fracture load measurements. Specimens were fixed in a three-piece metal die (second premolar, upper first molar, and second molar). The fracture test was tested using a semi-clinical experimental design under ambient laboratory conditions. The test was performed using the load compression mode applied occlusally on the surface of the splint at a rate of 0.5 mm/min until failure occurred. The maximum limit of fracture toughness was recorded in newtons (N).

**Figure 3:**
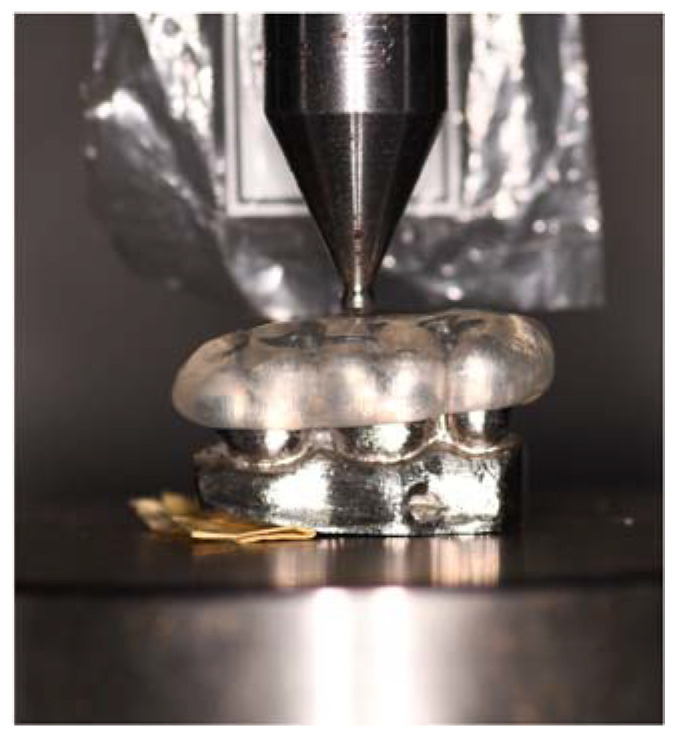
In vitro test.

**Figure 4:**
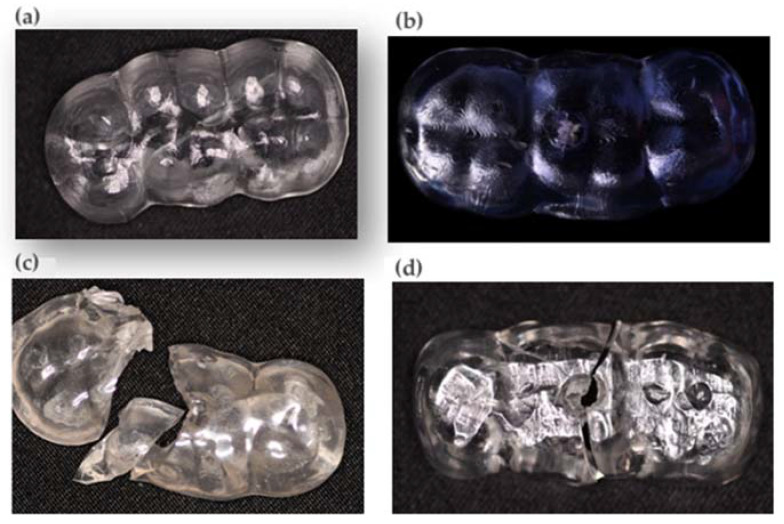
(**a**) Milled splint test. (**b**) Printed splint test (nightguard flex resin). (**c**) Printed splint test (resin splint). (**d**) Acrylic splint test.

**Figure 5:**
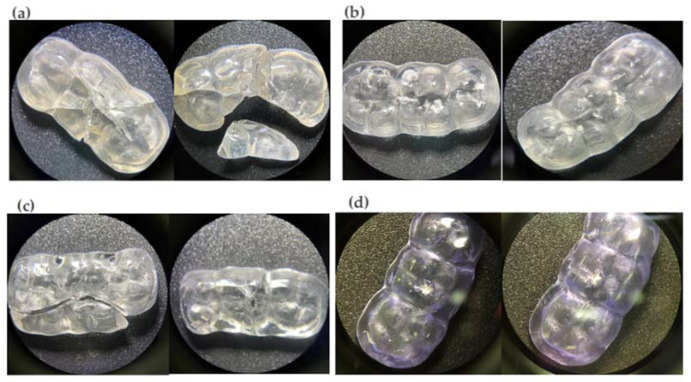
Microscopic analysis of (**a**) printed splint, (**b**) milled splint, (**c**) acrylic splint, and (**d**) printed splint (nightguard flex resin) tests.

A compression load was applied by a semi-hemispherical indenter (D = 3 mm) with a speed of 0.5 mm/min on the occlusal surface until fracture occurred (Figure 3 and Figure 4). Failure was defined as the moment when the load fell 5% below its maximum value. A preload of 10 N was applied. Fracture surface analysis was performed with the aid of a high-resolution stereo microscope (Nikon C-LEDS, Melville, NY, USA) to identify the fracture mode (Figure 5).

## 3. Statistical Analysis

The analyses were calculated with statistical software (SPSS V26; IBM Corp., Armonk, NY, USA). The assumptions of normality were verified with the Shapiro-Wilk test and homoscedasticity, or equality of variance, with Levene’s test. The three-way ANOVA test was used to determine differences between materials, and Tukey’s HSD test was used to specifically confirm the intergroup difference.

## 4. Results

Table 1 presents a descriptive analysis of the fracture resistance measurements obtained from the four splints (milled, printed, conventional, and flexible). 

Regarding the present data dispersion, the milled splint registered the lowest dispersion (coefficient variation (CV) = 5.9%), followed by the printed splint (CV = 6.7%), the conventional splint (CV = 7.0%), and the flexible splint, which showed a medium level of dispersion (CV = 14.5%). These results suggest that the conventional, printed, and milled splints presented greater precision between measurements. 

According to the research hypothesis proposed to determine differences between three populations, it is necessary to verify the assumptions of normality with the Shapiro-Wilk statistic and homoscedasticity, or equality of variance, with Levene’s test to select the appropriate statistical technique. According to the significance values (*p*-value) in Table 2, the fulfillment of both assumptions was evidenced since they are greater than the significance level, allowing us to reject the null hypothesis of normal distribution and the null hypothesis of homoscedasticity, thus selecting the ANOVA technique.

With the results of the analysis of variation observed in Table 3, the null hypothesis was rejected (F = 59.953, *p*-value < 0.05), showing that there were differences in the resistance to fracture. According to this result, a multiple comparison test was carried out using the Tukey HSD, which can be reviewed in the following table.

According to the results in Table 4, the null hypothesis of equality between types of material was rejected, proving that there were significant differences between the average fracture resistance of the printed splint and the conventional splint (Diff = 1107.77, *p*-value < 0.05); significant differences were also found between the average fracture resistance of the milled splint and the conventional splint (Diff = 1747.24, *p*-value < 0.05) and between the average fracture resistance of the conventional splint and the flexible splint (Diff = 1561.29, *p*-value < 0.05). Likewise, no differences were observed in the average fracture resistance using the milled splint or the flexible splint (Diff = 185.95, *p*-value > 0.05), and there was no difference in the average fracture resistance between the printed splint and the flexible splint (Diff = 453.52, *p*-value > 0.05).

From the fractographic analysis, it is observed that the 2 materials (printed splint (resin splint) and acrylic splint) of the 4 compared present a brittle fracture. Once the critical stress value is reached, brittle materials present unstable cracks; that is, they do not require an increase in stress for spontaneous crack propagation, and catastrophic failure occurs. In the milled splint, only one crack formation is observed despite having reached the maximum value of applied force without catastrophic damage in Table 5 (Figure 5).

## 5. Discussion

There is a growing trend of interest in analyzing the behavior and usefulness of materials and digital flow, especially in the areas of prosthetics, implantology, and maxillofacial surgery. In the area of temporomandibular disorders and specifically in the manufacture of occlusal splints, it is essential to study their mechanical behavior. The aim of this research was to establish whether there are differences in the mechanical behavior of occlusal splints depending on the material and method of manufacture. 

For this purpose, different materials for the production of splints were analyzed: conventional, milled, 3D printed, rigid, and flexible, and it was found that the milled materials were more resistant to fracture than the conventional ones, 3D printed, and flexible materials. Therefore, the null hypothesis that the manufacturing process of the different materials would not affect their mechanical properties was rejected. Within the materials selection criteria, according to ISO norms, national and international standards should be established with the objective of providing a catalog of minimum requirements and standardized testing techniques for materials. Splints do not have requirements or well-defined standards; however, in order to evaluate the materials of which they are composed, the materials can be studied according to the standards of those used in resin prostheses [9]. 

In a study developed by Lutz (2019), the fracture and wear resistance of printed, milled, and conventional splints were evaluated. The results of this study showed that the milled splints presented a higher fracture resistance with an initial resistance of 3398 ± 435 N, while the printed splints presented a value of 2286 + 499 N and the conventional ones of 2393 + 451 N. Additionally, in the wear simulation, the milled splints showed better behavior than the printed and conventional splints. Such results were given by the industrial manufacture of PMMA CAD/CAM blanks. On the contrary, for the production of conventional splints, even though it is performed in PMMA, it is a more vulnerable technique due to the influence of the operator, which would reduce the conversion rate of double bonds, the presence of bubbles, and the lack of homogeneity in the material.

With reference to the material for printed splints, the producer provides limited information about its chemical composition compared to the other materials for milled and conventional splints that possess PMMA. However, despite the loss of material volume observed in the printed material, the fracture toughness was not affected at the time of chewing simulation, showing good aging behavior, perhaps due to the homogeneity of the material, not only because of the chemical composition but also because of the processing by 3D printing, which makes the material recommended only for short-term use.

As mentioned above, it is important to consider the chemical composition of materials when determining the mechanical behavior of three-dimensional printed resins. Manufacturers rarely specify the exact composition of 3D printed resins, but it would be useful to have more information to better understand the chemical influence on the mechanical properties and to recommend the optimal material for treatment [21].

A study conducted by Schemeiser (2022) evaluated the wear of two samples of subtractive fabricated occlusal splint materials in comparison to three-dimensional printing by chewing simulation after 120,000 cycles to evaluate the different failures on the occlusal surfaces. The results showed a perforation for the milled sample and a fracture for the printed splint, and upon microscopic visualization, it could be observed that the milled samples exhibit a uniformly smooth structure with no perceptible difference, which is in agreement with the results described in the present investigation [22].

On the other hand, in an investigation conducted by Gibreel (2021), five CAD/CAM milled resin materials were evaluated, as were two conventional materials (self-curing and thermal polymerization), for flexural strength, elastic modulus, and fracture toughness [10].

As a conclusion of this study, the milled material (Temp Premiun Flex Transpa by Zirkonzahn) obtained higher strength values. Probably because, as they contain reduced levels of residual monomer, they absorb more energy and suffer a plastic deformation, which produces a deflection of the material. However, they do not fracture and show better resistance to crack propagation, confirming their ductility. In the present study, the material with the highest fracture toughness was also the CAD/CAM milled material with 3051.2 N, although the values were significantly higher than those of the aforementioned study, probably because a three-point bending test was used on a rectangular specimen [10], while in our study the specimens replicated the clinical conditions of the sample.

Another study by the same author evaluated the wear and surface hardness of nine materials, including PMMA (liquid/powder), PMMA discs, and light-curing resins, for the production of splints in conventional, milled, and printed forms, as well as the differences in wear and surface hardness. It was concluded that the PMMA-based splint materials showed surface hardness and uniformity in wear resistance, regardless of the manufacturing technology, while the 3D-printed light-curing resin material showed lower surface hardness and higher wear. This may be due to the layers that are deposited parallel to the direction of the load and the adhesion between successive layers [23]. In our study, the printed materials presented lower values than the milled ones; however, the flexural rigid material could present less possibility of fracture than a conventional one and also, for the same reasons stated above, less possibility of wear in the face of function or parafunction, although this should be corroborated by additional studies.

An investigation by Patzelt [24] in 2022, comparing conventional and digital workflows for the production of occlusal splints with regard to time efficiency, overall fit, and wear, reports that 15 splints were fabricated for both the conventional method (Probase Cold, Ivoclar Vivadent, Schaan, Liechtenstein) and the digital method with a PMMA guide disc (guide inCoris, Dentsply-Sirona), which were subjected to an occlusal wear test of the materials in a chewing simulator. In this study, it was found that there was less average wear for the conventionally fabricated splint material after 1.2 million load cycles; however, in terms of time, the digitally fabricated splints were recommended because there is a reduction in the number of steps during the fabrication. These results corroborate what was observed in our study and support what was described by Huettig (2017) and Orphan (2020), when mentioning that modern digital technology allows one to fabricate splints more efficiently and achieve shorter lead times, better accuracy, and improved design than when making a splint in the conventional method [5,24,25]. Insisting on this issue, for the occlusal wear test of the materials, it could be concluded that there was no impact of the different materials used in the conventional and digital workflows. The digital workflow for the production of occlusal splints leads to a better fit than the conventional workflow since the industrially manufactured PMMA has a higher density, higher degree of polymerization, higher homogeneity, and fewer pores in the splints produced from blanks compared to those manufactured using a conventional workflow [24].

Prpic [26] already stated that the differences were mainly due to the different chemical compositions, although the technology used also played a role. Mentioning the technology used, in the studies of Unkovskiy et al., the print orientation influenced the accuracy of the print [27]. Parameters printed along the Z-axis are particularly prone to inaccuracies. In addition, it was found that specimens with an orientation of 45° were the most accurate. The printed objects at the edges of the build platform are more prone to inaccuracies than those in the center. The 90° specimens with layer orientation parallel to axial loading showed superior flexural strength and flexural modulus. The use of a different curing unit is unlikely to affect the printing accuracy and flexural properties of the specimens. All these details were taken into account for the fabrication of the specimens in our study, as they were printed vertically with a layer thickness of 100 μm, and perhaps these features helped to improve the strength, agreeing with the study by Lutz [21], where milled splints had better fracture resistance than conventional and printed splints. These results, even though the methodology differed in terms of the fracture test, came from tests conducted on a single tooth, while in ours, at least three teeth were used to support the splint.

With reference to the layer thickness for 3D splint impressions, the manufacturer mentions that for rigid resin (Splint, SprintRay) the standard thickness should be 100 μm and for flexural rigid resin (Nightguard Flex) the suggested thickness should be 150 μm, noting that the layer thickness in this study did show differences since the rigid resin splint in our research was 100 μm and the flexural rigid splint was 150 μm. This means that the flexural rigid splint, being thicker, tends to be stronger, and the repeatability of the design was not affected. Similarly, in the study by Sabbah et al., the thickness of the 3D printing layer did not affect the repeatability or the surface roughness of the product. These findings can be explained by the standardization of the printing parameters, including specimen orientation, substrate number, substrate locations, resin used for printing, and post-processing methods, which were the same for all groups [28].

In view of all these results collected in the scientific evidence, it can be highlighted that the interesting characteristic determined in our study was the great resistance to fracture of the milled material (3051.2 N) in comparison with the 3D printed material, with the flexural rigid splint (1943.4 N) in third place, the printed splint (1489.9 N) following, and the lowest resistance to fracture being shown by the conventional splint with an average of 1303.9 N. This outcome is related to the study carried out by Cornwell, when he mentioned that the material with the highest resistance was the same material as the flexural rigid printed splint. In this academic work, hardness and wear were evaluated, but we could observe that the capacity of the flexural rigid material is maintained since its additive fabrication method generates adequate thicknesses for wear under in vitro conditions [29].

A systematic review by Lopez et al. (2023) analyzed several studies that evaluated the mechanical properties of printed, milled, and conventionally fabricated splints, concluding that milled resins showed improved mechanical performance in terms of hardness, wear resistance, flexural strength, flexural modulus, and fracture toughness compared to 3D printed and conventional resins, agreeing with the results of the present study in terms of the evaluated mechanical properties [30]. According to Reyes (2018), and agreeing with the methodology for the samples in his study, another important parameter to consider in the fabrication of occlusal splints is to design a flat and smooth surface without indentations. Such indentations can be eliminated in splints that are made by the CAD/CAM system since it is digitally possible to flatten the surface using software. On the other hand, removing indentations when splints are fabricated conventionally would require more work on the part of the dental technician or dentist [30,31]. These unfavorable mechanical properties may be the reason why printed splints have not been widely established so far. So as a possible solution and innovation, flexural rigid 3D printing resin was introduced. The material is characterized by moderate stiffness at room temperature and lower rigidity and viscoelastic behavior at body temperature. These characteristics could lead to higher toughness in the material. Therefore, the material could also be suitable for long-term use. Since there are no clinical data to support these assumptions, in the study by Herpel et al., they conducted a randomized controlled pilot trial where they concluded that 3D printed thermo-flexible occlusal splints could be a clinically viable alternative to milled splints for at least three months of clinical use, although their long-term use needs to be further investigated [31,32]. In our study, the flexural rigid splint had encouraging results, in addition to the resilience of the material, which would provide a better distribution of loads on the teeth to be protected and surrounding structures. Therefore, this material should claim a wider space in its indication for the fabrication of occlusal splints, as it could very well withstand the loads originating from occlusal contacts. Therefore, this consideration deserves at least an analysis of its effects. To compare the effects of functional contacts with parafunctional contacts, one study analyzed the amount of force applied to the teeth in kg/second/day during mastication and swallowing (in phonation, the teeth do not usually contact). It is estimated that in each chewing action, an average force of 26.59 kg is applied for 115 ms, i.e., 3.05 kg per chewing. Approximately 1800 chewing events occur in a day, so the value of total occlusal force/activity time is 5503.95 kg/day. As for swallowing, a person swallows about 146 times a day while eating. If a force of 30.12 kg is applied to the teeth for 522 seconds per swallow, a force of 2295.8 kg/day will be exerted. These result in a total force/time activity for chewing and swallowing of almost 7791.6 kg/day. High muscle activity refers to contractions greater than those required for swallowing, maintained for one second or more. One second means 39 units of activity. Normally, during sleep, 20 units per hour are produced, estimating for each unit a force of 36.24 kg/s. The 8-hour nocturnal activity is 5798.4 kg/s lower than the functional masticatory force. A bruxer can easily generate 60 units of activity per hour, or 17,395.2 kg/s per night, which is three times the functional daytime activity. This indicates that the force and duration of parafunctional tooth contacts are more harmful to the masticatory system than functional contacts [31,32,33]. Therefore, milled and 3D printed flexural rigid occlusal splints, according to our results, could withstand these values of the applied forces, even though the values differ from those reported commercially (Table 5).

Among the limitations of this study, its in vitro design would also require it to be carried out clinically, for example, using a volumetric surface analysis by means of digital superimposition. It would also be useful to include in the study variables such as material fabrication time, post-production costs, labor, and clinical behavior regarding the fracture resistance of these devices.

## 6. Conclusions

From the results obtained, it was concluded that the milled splints were the most resistant to fracture. As for the printed splints, the splints fabricated with flexural rigid resin adequately withstood the applied forces. Therefore, this new material can be recommended as a viable option for creating these devices. Although the results of this research indicated differences in the mechanical properties between CAD/CAM and conventional fabrication methods, the selection may also be influenced by the processing time since with a CAD/CAM system there is a significant reduction in production time. For that reason, the validity of the digital flow is ratified today with respect to the conventional manufacturing method.

## Figures and Tables

**Table 1:** Descriptive data on fracture resistance.

Material/Statistic	Acrylic Splint	Printed Splint (Resin Splint)	Flex Printed Splint (Nightguard Flex Resin)	Milled Splint
Mean	1303.9 N	1489.9 N	1943.4 N	3051.2 N
Standard deviation	90.7	99.8	281.21	179.07
Coefficient of variation	70%	6.7%	14.5%	5.9%
Minimum	1235.31	1378.6	1726.30	2884.66
Maximum	1406.82	1571.5	2261.08	3240.60

**Table 2:** Data verification.

Type of Splint	Normality Test Shapiro-Wilk Test		Levene Test
		*p*-Value	*p*-Value
Milled splint	0.893	0.364	0.191
Printed splint	0.904	0.399	
Acrylic splint	0.988	0.788	
Flex printed splint	0.284	0.501	

Note: Significance level: 5%. Equality of variances is assumed.

**Table 3:** Results of the analysis of variance.

Source of Variation	Sum of Squares	Quadratic Mean	F	*p*-Value
Between groups	5,525,106.1	1,841,702.03	56.953	<0.001
Within groups	258,696.2	32,337.01		
Total	5,783,802.3			

Note: Significance level: 5%. Dependent variable resistance to fracture (N).

**Table 4:** Results of the HSD Tukey test.

Type of Material		Difference of Mean	Sig.
Milled splint	Printed splint	1561.29 *	0.001
	Acrylic splint	1747.24 *	0.001
	Flex printed splint	1107.77 *	0.002
Acrylic splint	Flex printed splint	639.47 *	0.010

Note: dependent variable fracture resistance (N), HSD Tukey. * The difference in means is significant at the 0.05 level.

**Table 5:** Flexural strength of the materials used in this research.

Material	Resistance According to the Manufacturer	Resistance According to the Research
PMMA	≥100 MPa	26.31 MPa
Resin Splint	≥105 MPa	27.12 MPa
Nightguard Flex Resin	≥118 MPa	29.25 MPa
Disco ProArt CAD	≥100 MPa	46.99 MPa

## Data Availability

https://drive.google.com/drive/folders/1_3y01duHAqZ2ywsrtdOSF3ImSQ4twEf_ (accessed on 15 September 2023).

## References

[1] Liu F., Steinkeler A. (2013). Epidemiology, diagnosis, and treatment of temporomandibular disorders. Dent. Clin. N. Am..

[2] Dylina T. (2001). A common-sense approach to splint therapy. J. Prosthet. Dent..

[3] Boero R.P. (1989). The physiology of splint therapy: A literature review. Angle Orthod..

[4] Zhang S.-H., He K.-X., Lin C.-J., Liu X.-D., Wu L., Chen J., Rausch-Fan X. (2020). Efficacy of occlusal splints in the treatment of temporomandibular disorders: A systematic review of randomized controlled trials. Acta Odontol. Scand..

[5] Huettig F., Kustermann A., Kuscu E., Geis-Gerstorfer J., Spintzyk S. (2017). Polishability and wear resistance of splint material for oral appliances produced with conventional, subtractive, and additive manufacturing. J. Mech. Behav. Biomed. Mater..

[6] Xu X., He L., Zhu B., Li J., Li J. (2017). Advances in polymeric materials for dental applications. Polym. Chem..

[7] Wesemann C., Spies B.C., Schaefer D., Adali U., Beuer F., Pieralli S. (2021). Accuracy and its impact on fit of injection molded, milled and additively manufactured occlusal splints. J. Mech. Behav. Biomed. Mater..

[8] Mohammed A., Rahman Q., Uddin W. (2016). Efficacy of the acrylic splint in the treatment of the internal disorder of the temporomandibular joint. Bangladesh Med. Res..

[9] Berli C., Thieringer F.M., Sharma N., Müller J.A., Dedem P., Fischer J., Rohr N. (2020). Comparing the mechanical properties of pressed, milled, and 3D-printed resins for occlusal devices. J. Prosthet. Dent..

[10] Gibreel M., Perea-Lowery L., Vallittu P.K., Lassila L. (2021). Characterization of occlusal splint materials: CAD-CAM versus conventional resins. J. Mech. Behav. Biomed. Mater..

[11] Venezia P., Muzio LL O., Furia CD E., Torsello F. (2019). Digital manufacturing of occlusal splint: From intraoral scanning to 3D printing. J. Osseointegration.

[12] Rekow E.D. (2020). Digital dentistry: The new state of the art—Is it disruptive or destructive?. Dent. Mater..

[13] Msallem B., Sharma N., Cao S., Halbeisen F.S., Zeilhofer H.-F., Thieringer F.M. (2020). Evaluation of the dimensional accuracy of 3D-printed anatomical mandibular models using FFF, SLA, SLS, MJ, and BJ printing technology. J. Clin. Med..

[14] Dawood A., Marti B.M., Sauret-Jackson V., Darwood A. (2015). 3D printing in dentistry. Br. Dent. J..

[15] Etemad-Shahidi Y., Qallandar O.B., Evenden J., Alifui-Segbaya F., Ahmed K.E. (2020). Accuracy of 3-dimensionally printed full-arch dental models: A systematic review. J. Clin. Med..

[16] Shaikh S.S., Nahar P., Shaikh S.Y., Sayed A.J., Ali Habibullah M. (2021). Current perspectives of 3D printing in dental applications. Braz. Dent. Sci..

[17] Wang X., Shujaat S., Shaheen E., Jacobs R. (2021). Accuracy of desktop versus professional 3D printers for maxillofacial model production. A systematic review and meta-analysis. J. Dent..

[18] Upadhyay A., Khayambashi P., Farooq I., Sabri H. (2021). Dental 3D printing: Transferring the art from labs to clinics. Polymers.

[19] Yau H.T., Yang T.J., Lin Y.K. (2016). Comparison of 3D printing and 5-axis milling for the production of electronic dental models from intraoral scanning. Comput. Des. Appl..

[20] Zafar M.S. (2020). Prosthodontic applications of polymethyl methacrylate (PMMA): An update. Polymers.

[21] Lutz A.-M., Hampe R., Roos M., Lümkemann N., Eichberger M., Stawarczyk B. (2019). Fracture resistance and 2-body wear of 3-dimensional–printed occlusal devices. J. Prosthet. Dent..

[22] Schmeiser F., Baumert U., Stawarczyk B. (2022). Two-body wear of occlusal splint materials from subtractive computer-aided manufacturing and three-dimensional printing. Clin. Oral Investig..

[23] Gibreel M., Perea-lowery L., Vallittu P.K., Garoushi S. (2022). Two-body wear and surface hardness of occlusal splint materials. J. Dent. Mater..

[24] Patzelt S.B.M., Krügel M., Wesemann C., Pieralli S., Nold J., Spies B.C., Vach K., Kohal R.-J. (2022). In Vitro Time Efficiency, Fit, and Wear of Conventionally-versus Digitally-Fabricated Occlusal Splints. Materials.

[25] Rungrojwittayakul O., Kan J.Y., Shiozaki K., Swamidass R.S., Goodacre B.J., Goodacre C.J., Lozada J.L. (2020). Accuracy of 3DPrinted Models Created by Two Technologies of Printers with Different Designs of Model Base. J. Prosthodont..

[26] Prpic V., Slacanin I., Schauperl Z., Catic A., Dulcic N., Cimic S. (2019). A study of the flexural strength and surface hardness of different materials and technologies for occlusal device fabrication. J. Prosthet. Dent..

[27] Unkovskiy A., Bui P.H.-B., Schille C., Geis-Gerstorfer J., Huettig F., Spintzyk S. (2018). Objects build orientation, positioning, and curing influence dimensional accuracy and flexural properties of stereolithographically printed resin. Dent. Mater..

[28] Sabbah A., Romanos G., Delgado-Ruiz R. (2021). Impact of layer thickness and storage time on the properties of 3D-printed dental dies. Materials.

[29] Cornwell C. (2022). Simulated Nighttime Grinding of 3D Printed Night Guards vs Lab Manufactured Night Guards. Open Access J. Dent. Sci..

[30] De Paula Lopez V., Dias Corpa Tardelli J., Botelho A.L., Marcondes Agnelli J.A., Cândido Dos Reis A. (2023). Mechanical performance of 3-dimensionally printed resins compared with conventional and milled resins for the manufacture of occlusal devices: A systematic review. J. Prosthet. Dent..

[31] Reyes-Sevilla M., Kuijs R.H., Werner A., Kleverlaan C.J., Lobbezoo F. (2018). Comparison of wear between occlusal splint materials and resin composite materials. J. Oral Rehabil..

[32] Herpel C., Kykal J., Rues S., Schwindling F.S., Rammelsberg P., Eberhard L. (2023). Thermo-flexible resin for the 3D printing of occlusal splints: A randomized pilot trial. J. Dent..

[33] Okeson J.P. (2019). Management of Temporomandibular Disorders and Occlusion.

